# The simultaneous perception of auditory–tactile stimuli in voluntary movement

**DOI:** 10.3389/fpsyg.2015.01429

**Published:** 2015-09-24

**Authors:** Qiao Hao, Taiki Ogata, Ken-ichiro Ogawa, Jinhwan Kwon, Yoshihiro Miyake

**Affiliations:** ^1^Department of Computational Intelligence and Systems Science, Tokyo Institute of TechnologyYokohama, Japan; ^2^Research into Artifacts, Center for Engineering (RACE), The University of TokyoKashiwa, Japan

**Keywords:** voluntary movement, temporal simultaneity, auditory–tactile stimuli, temporal order judgment, efference copy

## Abstract

The simultaneous perception of multimodal information in the environment during voluntary movement is very important for effective reactions to the environment. Previous studies have found that voluntary movement affects the simultaneous perception of auditory and tactile stimuli. However, the results of these experiments are not completely consistent, and the differences may be attributable to methodological differences in the previous studies. In this study, we investigated the effect of voluntary movement on the simultaneous perception of auditory and tactile stimuli using a temporal order judgment task with voluntary movement, involuntary movement, and no movement. To eliminate the potential effect of stimulus predictability and the effect of spatial information associated with large-scale movement in the previous studies, we randomized the interval between the start of movement and the first stimulus, and used small-scale movement. As a result, the point of subjective simultaneity (PSS) during voluntary movement shifted from the tactile stimulus being first during involuntary movement or no movement to the auditory stimulus being first. The just noticeable difference (JND), an indicator of temporal resolution, did not differ across the three conditions. These results indicate that voluntary movement itself affects the PSS in auditory–tactile simultaneous perception, but it does not influence the JND. In the discussion of these results, we suggest that simultaneous perception may be affected by the efference copy.

## Introduction

When people type quickly on a computer keyboard they usually integrate visual, auditory, and tactile information to ensure successful performance. For efficient interactions with the environment or other people, the simultaneous perception of multimodal information is important during voluntary movement, and determines the timing of multimodal events. Many previous studies have focused on the simultaneous perception of multimodal information under static experimental conditions during which participants remain immobile. However, how the timing of multimodal events is determined during voluntary movements remains largely a mystery. Although voluntary movement has been found to compress or dilate subjective time under certain circumstances (Yarrow et al., 2001; Morrone et al., 2005), current knowledge about the effect of voluntary movement on auditory–tactile simultaneous perception is still unsettled. In particular, it is unclear whether voluntary movement or proprioceptive information following a movement affects the simultaneous perception of auditory and tactile stimuli.

To investigate the fundamental characteristics of simultaneous perception, simultaneity judgment (SJ) tasks (Schneider and Bavelier, 2003; Zampini et al., 2005a) or TOJ tasks (Mitrani et al., 1986; Spence et al., 2001; Zampini et al., 2003; Miller and Schwarz, 2006; Cardoso-Leite et al., 2007; Boenke et al., 2009; Van Eijk et al., 2009; Kwon et al., 2014) are often used. In a SJ task, two stimuli are presented at various SOAs and the participants are asked to indicate whether the two stimuli are simultaneous or not. In a TOJ task, the participants are required to judge the temporal order of the two stimuli. These tasks have revealed that people tend to perceive different modal stimuli as occurring simultaneously when they are presented with a short lag (Slutsky and Recanzone, 2001; Lewald and Guski, 2003; Kayser et al., 2008; Shi et al., 2008; Nishi et al., 2014). More specifically, the PSS differs from the point of physical simultaneity. Furthermore, temporal resolution is usually evaluated by JND, which represents difference threshold of SJ or TOJ task, with a lower JND indicating higher temporal resolution, and vice versa. JNDs differ for different combinations of multimodal information types (Keetels and Vroomen, 2005, 2008; Zampini et al., 2005b).

Some previous studies have shown that voluntary movements affect the PSSs and/or JNDs between visual–tactile stimuli (Vogels, 2004; Shi et al., 2008) and between auditory–tactile stimuli (Kitagawa et al., 2009; Frissen et al., 2012; Nishi et al., 2014) in SJ and TOJ tasks compared with conditions without voluntary movement. To investigate the effect of voluntary movement on simultaneous perception, the effect of proprioceptive sensation attending the movement must be separated from that of voluntary movement. If PSS and/or JND changes are observed even when the proprioceptive information effect is excluded, we can say that the voluntary movement itself has some influence on simultaneous perception. Therefore, voluntary, involuntary, and no movement conditions were used in previous studies (Kitagawa et al., 2009; Frissen et al., 2012; Nishi et al., 2014). Because a device moved the participants’ body parts in the involuntary movement condition in the previous studies, the involuntary movement was attended by proprioceptive information. Therefore, the comparison between the involuntary and no movement conditions showed the effect of the proprioceptive information, and the comparison between the voluntary and involuntary movement conditions revealed the effect of voluntary movement exclusive of proprioceptive information.

However, those investigations of the effect of voluntary movements on the PSSs and JNDs in the auditory–tactile TOJ tasks reported contradictory results (**Table 1**, Effect on PSS and Effect on JND rows). Kitagawa et al. (2009) found that voluntary movement did not affect the PSS, whereas Nishi et al. (2014) found that voluntary movement caused the PSS to be associated with a preceding auditory stimulus. In addition, Frissen et al. (2012) found that involuntary movement caused the PSS to be associated with a preceding tactile stimulus. On the other hand, although Frissen et al. (2012) observed no effect of voluntary movement on the JND, Kitagawa et al. (2009) and Nishi et al. (2014) reported that voluntary movement improved the JND. These differing results may have been caused by unexpected effects associated with the different experimental methods used in the previous studies, such as the predictability of the stimulus and the amount of movement (**Table 1**, Predictability of the stimulus and Moving body part rows). For instance, a predictable stimulus could directly improve the JND (Petrini et al., 2009; Yokoyama et al., 2009; Vroomen and Stekelenburg, 2010). The spatial information in large-scale movement could obscure the effect of involuntary movement on the PSS.

**Table 1:** Comparison of methods and results among three previous studies of the effect of voluntary movement on auditory–tactile TOJ tasks.

Study	Kitagawa et al., 2009				Frissen et al., 2012			Nishi et al., 2014		
Conditions of movement	Vol	Inv	Pr	No	Vol	Inv	No	Vol	Inv	No
Predictability of the stimulus	Predictable				Not predictable			Predictable		
Moving body part	Right index finger				Forearm, hand, and finger			Right index finger		
Effect on PSS	N.S.				A shifted to T (Inv. movement)			T shifted to A (Vol. movement)		
Effect on JND	L (Vol. movement)				N.S.			L (Vol. movement)		

*“Vol,” “Inv,” “Pr,” and “No” indicate the voluntary, involuntary, predictable, and no movement conditions. For the effect on PSS, “N.S.” means no significant difference. “A shifted to T” means the PSS in the involuntary movement condition shifted from the auditory stimulus first as in the no movement condition to the tactile stimulus first, where “A” and “T” indicate the auditory and tactile stimuli. “T shifted to A” means the PSS in the voluntary movement condition shifted from the tactile stimulus first as in the no movement condition to the auditory stimulus first. For the Effect on JND, “L” and “H” indicate that the JND was improved (lower JND) or impaired (higher JND) by voluntary movement*.

Kitagawa et al. (2009) conducted the TOJ task under four conditions: voluntary, involuntary, predictable, and no movement (**Table 1**, Conditions of movement row). The participants pressed the button voluntarily and involuntarily with their fingers in the voluntary and involuntary movement conditions, respectively. The predictable condition was designed to enable participants to predict the occurrence of the stimulus in the TOJ task. The authors concluded that voluntary movement improved the participants’ JND, because there was no improvement in the JNDs of the involuntary, predictable, and no movement conditions. However, in Kitagawa et al.’s (2009) procedure, tactile stimulation was generated as a result of voluntary finger movement. This effect induced the participants to predict the onset of the tactile stimulus (**Table 1**, Predictability of the stimulus row), and improved the JND of the voluntary movement condition (**Table 1**, Effect on JND row). Nishi et al. (2014) conducted the TOJ task under three conditions: voluntary, involuntary, and no movement (**Table 1**, Conditions of movement row). The authors used a device that presented tactile stimulus during voluntary finger movement to solve the problem in Kitagawa et al.’s (2009) procedure. Nevertheless, this prediction effect on the improvement in the JND associated with voluntary movement also occurred in Nishi et al.’s (2014) study (**Table 1**, Effect on JND and Predictability of the stimulus rows), because the tactile stimulus was always presented 500 ms after the finger movement. It was easier to predict the stimulus onset in the voluntary movement condition.

This predictability of stimulus onset did not appear in the Frissen et al.’s (2012) study. The authors used a device that presented the tactile stimulus for the TOJ task at random interval in the voluntary movement condition, to prevent the stimulus predictability (**Table 1**, Predictability of the stimulus row). As a result, Frissen et al. (2012) reported that voluntary movement did not affect the JND (**Table 1**, Effect on JND row). This result suggests that the predictability of the stimulus improved the JNDs both in the Kitagawa et al.’s (2009) and Nishi et al.’s (2014) studies. On the other hand, Frissen et al. (2012) reported that the tactile stimulus occurring first was perceived as the PSS in the involuntary movement condition (**Table 1**, Effect on PSS row). However, the spatial information in large-scale movement (**Table 1**, Moving body part row) could have obscured the effect of movement on the PSS in Frissen et al.’s (2012) study. The large-scale movement could lead to a tactile version of a flash-lag effect (FLE; Kitagawa et al., 2005). In this phenomenon, observers perceived a flash lag behind a spatially aligned moving stimulus (Nijhawan, 2002).

Therefore, the aim of the present study was to investigate whether only voluntary movement alone affects the simultaneous perception of auditory and tactile stimuli, that is, independent of the effects of stimulus predictability and the spatial information inherent in large-scale movement (which were thought to be the causes of the divergent results in previous studies). We hypothesized that the PSS would shift from the tactile stimulus first in the involuntary movement or no movement condition to the auditory stimulus first in the voluntary movement condition. Thus, we randomized the interval between the start of movement and the first stimulus to prevent the participants from predicting the stimulus onset. In addition, we used small-scale movement to minimize the effect of spatial information on perceived simultaneity.

## Materials and Methods

### Participants

Eighteen participants (three females and 15 males, mean age: 23 years, age range: 21–28 years) completed the experiment. All of the participants were right-handed, with normal auditory thresholds and senses of touch, and they did not exhibit any difficulty moving their right index fingers. Informed consent was obtained in writing from all the participants prior to their participation in the experiment. The participants were paid for their participation, and the experiment was approved by the ethics committee of the Tokyo Institute of Technology.

### Apparatus and Stimuli

The auditory stimulus was a sinusoidal wave sound (2000 Hz, 50 dB, 10 ms) presented in both ears simultaneously via earphones (Radius HP-RHF41; Machida, Tokyo, Japan). The tactile stimulus was an impulse force (5 N, 10 ms, rectangular pulse) provided by a PHANTOM Desktop haptic device (SensAble Technologies, Woburn, MA, USA) and orthogonal to the finger movement. The 10 ms duration for auditory and tactile stimuli was selected to avoid a problem of the procedure in the Frissen et al.’s (2012) study. In that study, the duration of the auditory stimulus (100 ms) was considerably longer than that of the tactile stimulus (10 ms). Stimulus duration has been found to create an attractor effect on the PSS in audiovisual TOJ task (Boenke et al., 2009). In other words, with increasing stimulus duration, positive PSSs shift toward negative values (because the visual stimulus must precede the auditory stimulus for simultaneous perception), and negative PSSs shift toward positive values. Hence, we used the same duration for the two stimuli. The timing of the two presentations and the movement of the device were controlled to within an error margin of 1 ms. These sensory stimulation systems were operated by computer programs installed on a PC workstation (HP xw4600/CT; Hewlett-Packard, Palo Alto, CA, USA), and were developed with the Open Haptics software development toolkit (SensAble Technologies) on the Microsoft Visual C++ 2008 platform (Microsoft, Redmond, WA, USA).

### Task and Conditions

For the TOJ task, auditory–tactile stimulus pairs were presented to participants with varying SOAs (intervals between the within-pair onsets of the auditory and tactile stimuli), and the participants judged the temporal order of the two stimuli. The SOAs were ±240, ±120, ±60, ±30, and 0 ms (where the positive values indicate that the auditory stimulus was presented before the tactile stimulus, and vice versa). We chose these SOAs to improve the procedures in the Frissen et al.’s (2012) study. In that study, they used a 75 ms increment between their SOAs (300, 225, 150, 75, and 0 ms), which is a little larger than the increments used in previous auditory–tactile integration studies (Zampini et al., 2005b; Fujisaki and Nishida, 2009). Thus, we used a smaller increment for our SOAs.

**FIGURE 1:**
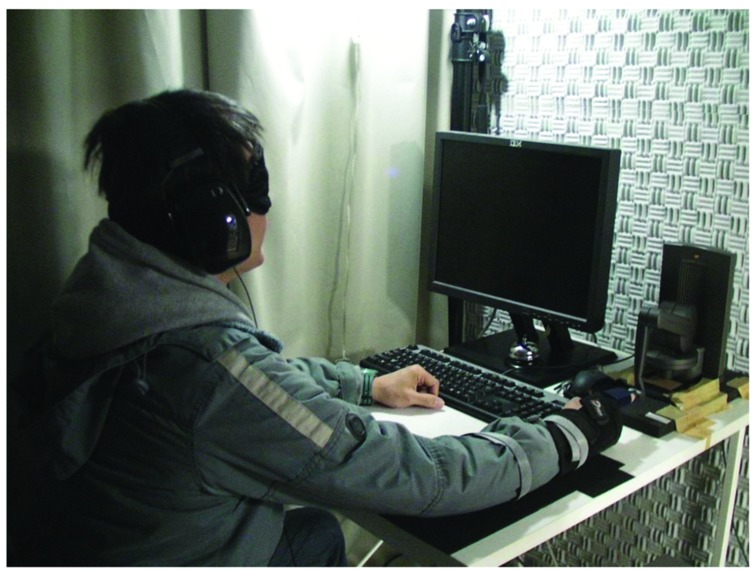
**Experimental environment**.

There were three conditions in this experiment: voluntary, involuntary, and no movement. The involuntary movement trajectory was reproduced from voluntary movement data collected in the preliminary experiments. The mean rate of movement of the participants’ fingers was 81.08 mm/s (*SD* = 7.33) in the voluntary movement condition and ∼78.23 mm/s (*SD* = 1.44) in the involuntary movement condition (as guided by the haptic device). The participants were seated in a darkened, sound-attenuated room in front of the stimulation systems, with the palmar side of their right index fingers held on the haptic device. They also wore sound-insulating earmuffs over their earphones and an eye mask to eliminate the confounding effect of visual stimuli during the experiment (**Figure 1**). In each condition, the participants were asked to indicate the temporal order of the auditory and tactile stimuli by using the Z and X keys on a keyboard. The Z indicated that the auditory stimulus occurred first and the X indicated that the tactile stimulus occurred first.

### Procedure

#### Voluntary Movement Condition

**FIGURE 2:**
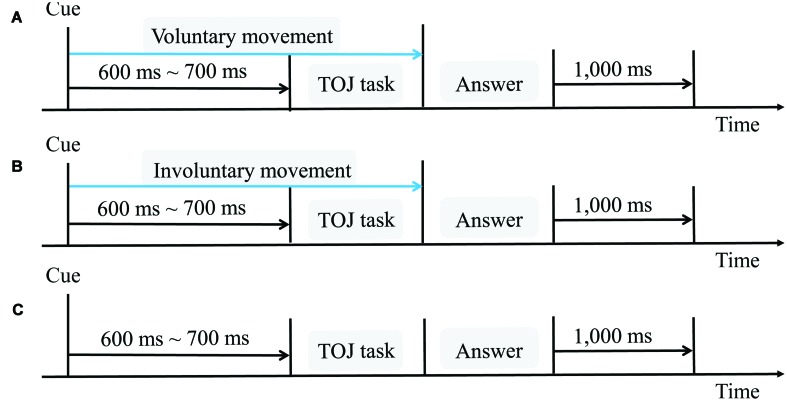
**Schematic flow chart for one trial in each of the three conditions. (A)** Voluntary movement condition, in which participants voluntarily started to move their right index fingers; **(B)** involuntary movement condition, in which the haptic device moved the participants’ right index fingers; **(C)** no movement condition. The interval between the cue and the TOJ task was randomly set from 600 to 700 ms. The interval between trials was 1000 ms.

For each trial (**Figure 2A**), the participants voluntarily and naturally began to move their right index fingers from right to left at their own pace. As they did, a cue sound (distinct from the target auditory stimulus) indicated that the TOJ task was forthcoming. The first stimulus (either tactile or auditory) was then presented with a random delay of 600–700 ms after the cue sound onset. The second stimulus (auditory or tactile, whichever was not presented first) followed the first stimulus, offset by one of the nine SOAs previously mentioned. The participants then indicated which stimulus occurred first using a two-alternative forced-choice test (as described above). If the participants did not move at a speed of 50–110 mm/s, they were given one more trial, randomly chosen from the remaining trials.

#### Involuntary Movement Condition

Similar to the voluntary movement condition, the haptic device randomly started to move the participants’ right index fingers from right to left for 500 to 1000 ms, to reproduce the variance in the onsets of voluntary movements in the preliminary experiments. The procedure for evaluating the temporal order of the two stimuli and the SOA values were the same as in the voluntary movement condition. A speed of 76 mm/s for the finger movement was set for each trial (**Figure 2B**), because this was considered to be a comfortable speed and representative of normal surface exploration.

#### No Movement Condition

The participants in the no movement condition remained stationary throughout each trial, with the palmar side of their right index fingers held on the haptic device (**Figure 2C**). The first stimulus (either tactile or auditory) was presented with a random delay (600–700 ms) after the cue sound onset. The presentation of the second stimulus and the procedure for evaluating the temporal order of the two stimuli were the same as in the voluntary and involuntary movement conditions. We used the 600–700 ms interval between the cue sound onset and the first stimulus to improve the procedure used in Nishi et al.’s (2014) study. In that study, the interval between the cue sound onset and the first stimulus was 1800–3300 ms in the no movement condition, whereas it was 500 ms between the cue sound onset (or the start of movement) and the tactile stimulus for all trials in the voluntary and involuntary movement conditions. This may have affected the comparisons among the conditions, because the different cue-target intervals activate distinct brain areas (Coull et al., 2000), affect temporal discrimination, and influence early perceptual processing (Sanders and Astheimer, 2008).

Each participant completed three blocks of trials in each of the conditions in the present experiment. The conditions were presented in a random order, and the participants were blind to the order of the conditions. Each block consisted of 45 trials, comprising five trials for each SOA, randomly selected from the following values: ±240, ±120, ±60, ±30, and 0 ms. Thus, each participant completed 405 trials. The interval between trials was 1000 ms in each condition, and white noise was played in the background to effectively mask any sounds made by the haptic device. It took ∼5 min for the participants to complete one block of trials. They were given several minutes of rest between blocks, according to their preferences. The order of the conditions was counterbalanced, and the entire procedure took ∼2 h. To accustom the participants in the voluntary movement condition to the appropriate finger speeds, they each completed a practice run of ten trials in which only the tactile stimulus was presented. To eliminate this compound effect (e.g., sensitization of the tactile channel), the participants were given 2–3 min of rest before each block of trials in the voluntary movement condition. Additionally, the participants were asked to pay constant attention to the tactile stimulus to control for the prior entry effect (Shore et al., 2001; Spence et al., 2001; Kitagawa et al., 2005; Zampini et al., 2005c), which facilitates the processing of an attended stimulus relative to an unattended stimulus.

For each trial in the practice sessions, the participants were asked to close their eyes and judge the order of the two stimuli and then open their eyes to see the feedback on the computer screen. With no information about the forthcoming condition, they completed 45, 20, and 20 trials in the voluntary, involuntary, and no movement conditions, respectively. The orders of the trials were counterbalanced, and the SOA was randomly chosen from ±240, ±120, and ±60 ms. In addition, the short interval (600–700 ms) between the onset of the movement and the TOJ task may have produced a strong interaction between the tactile signals elicited by the onset of the movement and by the tactile stimulus in the TOJ task. Thus, there appears to be a risk that the results of this study may be unclear. In fact, movement onset has been found to impair the temporal order threshold immediately following operant actions, but then reverts in the later action-effect interval (450–850 ms; Wenke and Haggard, 2009). Furthermore, the potential strong interaction did not appear to affect the tactile TOJ tasks in studies by Hermosillo et al. (2011) or Nishikawa et al. (2015), in which they used short intervals between the onset of movements and TOJ tasks. Therefore, the possibility of a strong interaction does not threaten the results of this study.

### Data Analysis

**FIGURE 3:**
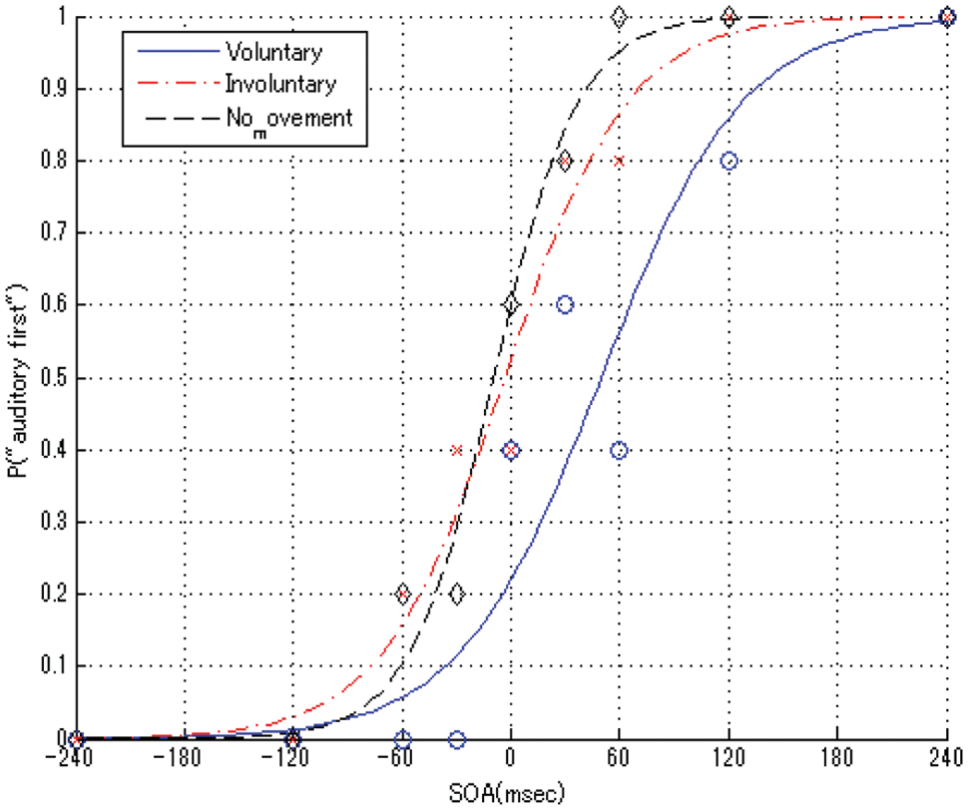
**Average psychometric functions among all blocks in the voluntary, involuntary, and no movement conditions for one participant**. Positive SOA values mean that the auditory stimulus was presented before the tactile stimulus, and vice versa.

We used MATLAB Statistics Toolbox (MathWorks, Natick, MA, USA) for the statistical regression calculations and graphic representation of the results. First, we calculated for each SOA the proportion of the answers, in which the auditory stimulus was perceived first. Then, logistic regressions were conducted using a generalized linear model with the ratio data for each condition. Psychometric curves were fitted to the distribution of the mean TOJ data for the voluntary, involuntary, and no movement conditions, as shown in **Figure 3**.

The values of the PSS and JND were calculated for each participant in the regression analysis based on three equations (Finney, 1952):

(1) $$y=\frac{1}{1\,\;+\;\;e^{\frac{\left(\alpha -x\right)}{\beta }}}\,\;\;\;\;\;\;\;\;\;\;\;\;\;\;\;\;\;\;\;\;\;\;\;\;\;\;\;\;\;\;$$

(2) pass=α⁢                             

(3) $$JND=\frac{x_{75}\,\;-\;x_{25}}{2}=\beta \;\;\;\log\,\;\;\;3\,\;\;\;\;\;\;\;\;\;\;\;\;\;\;\;\;\;\;\;\;\;\;\;\;\;\;\;\;\;\;$$

Here, *α* represents the estimated PSS, *x* denotes the SOA, *β* is related to the JND, and *x_p_* represents the SOA with *p* as the percent of “auditory first” responses. Then, a statistical analysis of the data was conducted to obtain the mean and standard error values for each condition.

## Results

**FIGURE 4:**
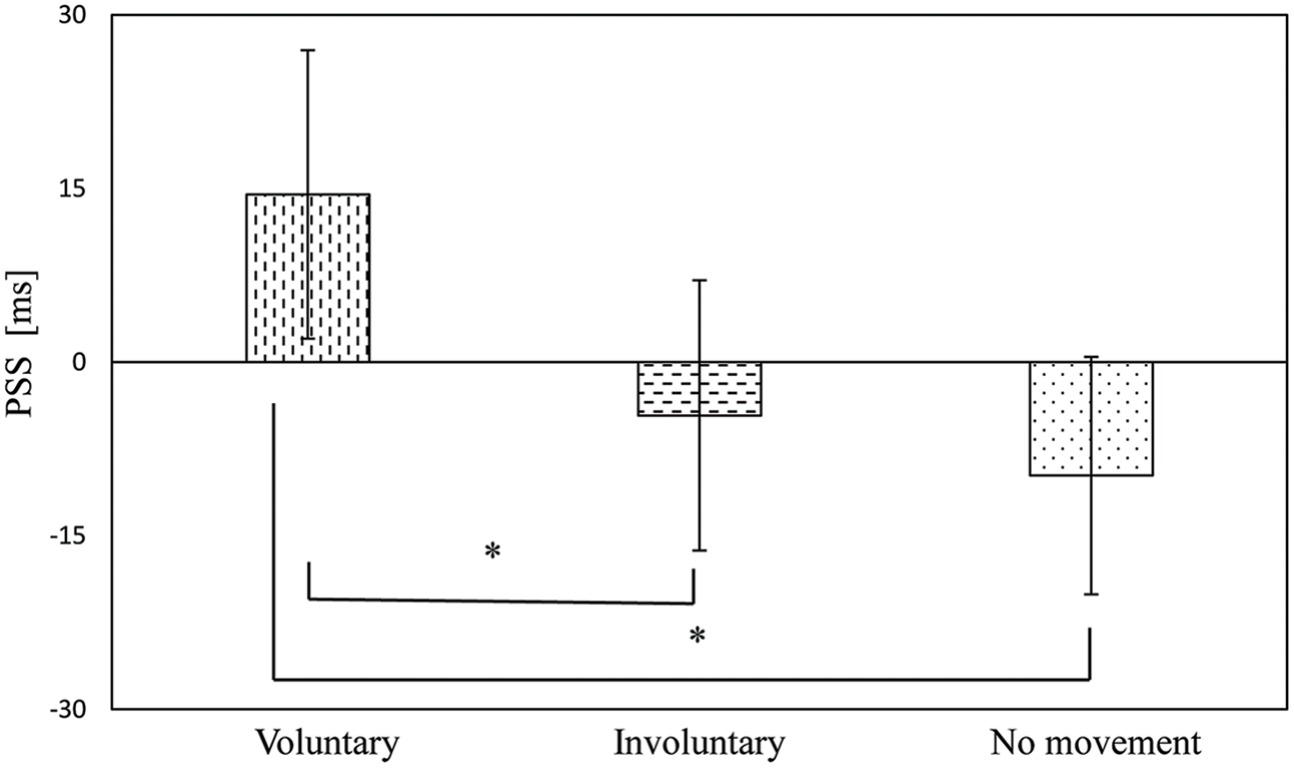
**Point of subjective simultaneity results in the voluntary, involuntary, and no movement conditions**. Error bars represent standard errors. ^∗^*p <* 0.01.

The PSSs of the voluntary, involuntary, and no movement conditions were 14.5 ms (*SE* = 12.5), –4.6 ms (*SE* = 11.7), and –9.8 ms (*SE* = 10.3), respectively, as shown in **Figure 4**. A one-way repeated measures analysis of variance (ANOVA) with movement condition as a factor showed a significant effect [*F*(2,34) = 12.74, *p* < 0.001]. Subsequently, Bonferroni–Holm paired *t*-tests revealed significant differences between the voluntary and involuntary movement conditions (*p* = 0.001), and between the voluntary and no movement conditions (*p* = 0.008). There was no significant difference between the involuntary and no movement conditions (*p* = 0.70), as shown in **Figure 4**. The magnitude of the effect size in the PSS (η^2^ = 0.43) was large (Cohen, 1988).

**FIGURE 5:**
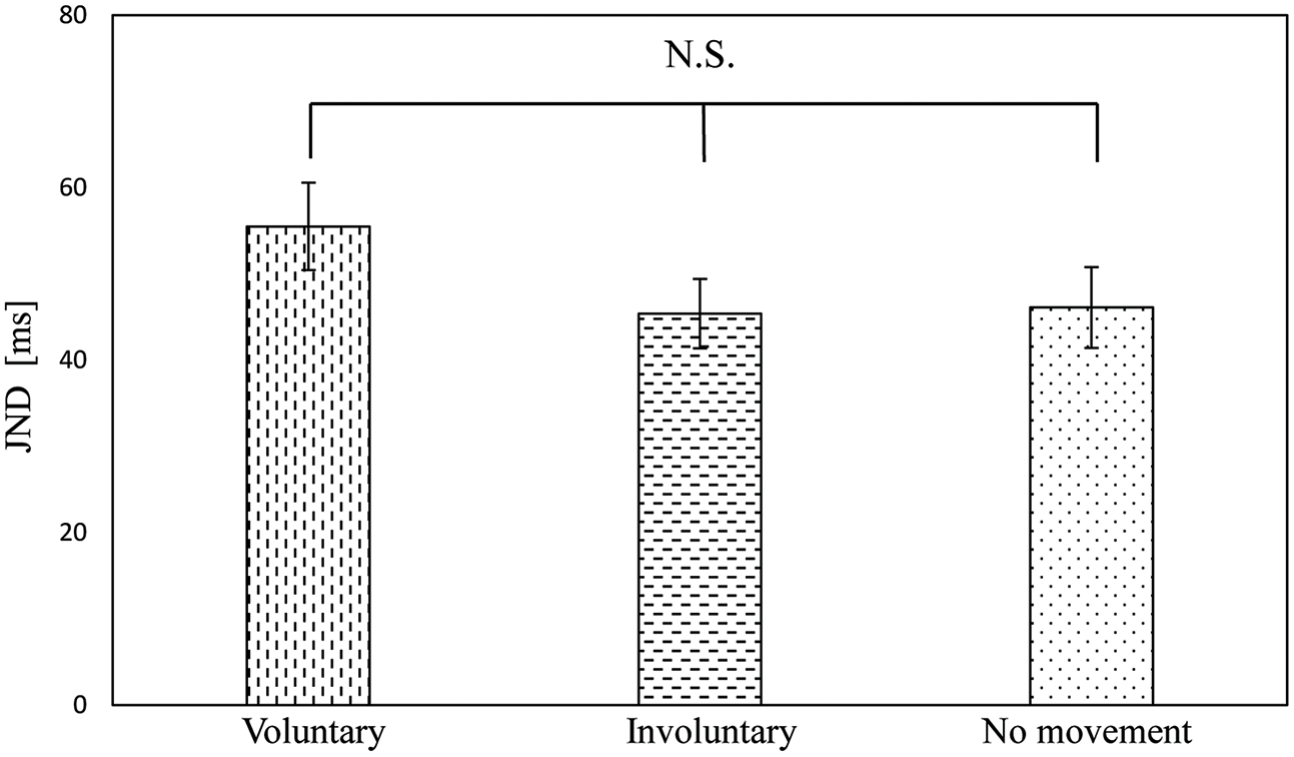
**Just noticeable difference results in voluntary, involuntary, and no movement conditions**. Error bars represent standard errors.

The JNDs of the voluntary, involuntary, and no movement conditions were 55.5 ms (*SE* = 5.1), 45.4 ms (*SE* = 4.0), and 46.1 ms (*SE* = 4.7), respectively. A one-way repeated measures ANOVA with movement condition as a factor was not significant [*F*(2,34) = 2.28, *p* = 0.12], with *p* = 0.26 between the voluntary and involuntary movement conditions, *p* = 0.30 between the voluntary and no movement conditions, and *p* = 1.0 between the involuntary and no movement conditions, as shown in **Figure 5**. The magnitude of the effect size for the JND (η^2^ = 0.12) was medium (Cohen, 1988).

## Discussion

The aim of this study was to isolate the potential impacts of methodological differences on the results of previous studies and investigate the effect of voluntary movement on the simultaneous perception of auditory and tactile stimuli in a TOJ task. In the present study, the potential effect of predictability on JNDs in Kitagawa et al.’s (2009) and Nishi et al.’s (2014) studies was removed by randomizing the interval between the start of movement and the first stimulus in the voluntary movement condition. Furthermore, we minimized the potential effect of the spatial information associated with large-scale movement on the PSS of involuntary movement condition (which was a problem in the Frissen et al.’s (2012) study) by using small-scale movement.

The results of this study replicated the effect of voluntary movement on the PSS (Nishi et al., 2014) and the JND (Frissen et al., 2012) in previous studies. In this study, we found that there was a significant shift in the PSS of the voluntary movement condition relative to the PSS of the involuntary and no movement conditions. There was no significant difference in the PSS between the involuntary and no movement conditions. The JND was not influenced by voluntary movement compared with the other two conditions. We discuss these differences in more detail below.

### Effect of Voluntary Movement on PSS

**Table 2** shows the PSS results of the previous and present studies. The PSS shift associated with involuntary movement in the Frissen et al.’s (2012) and Nishi et al.’s (2014) studies was not observed in the present study (Inv–No column). This result suggests that in the Frissen et al.’s (2012) study, the spatial information of the large-scale movement significantly caused the PSS shift in the involuntary movement condition because the present study minimized the effect of spatial information in the involuntary movement condition. In addition, the lack of short-range SOAs in Frissen et al.’s (2012) study may conceal the difference between voluntary and no movement conditions (see Materials and Methods; Vol–No column). The different stimulus durations would also partially confound the interpretation of the PSSs in their results (see Materials and Methods). This result also suggests that in the Nishi et al.’s (2014) study, the PSS shift associated with involuntary movement was caused by the different intervals, which were between the start of movement and the tactile stimulus in the involuntary movement condition, and between the cue sound onset and the first stimulus in the no movement condition, respectively (see Materials and Methods). This effect caused by different intervals did not occurred in the present study, because we used the same interval between cue sound onset and the first stimulus throughout in the three conditions. The reasoning for this is as follows. First, the long cue-target intervals activate the areas of the brain involved in motor preparation, which are distinct from those activated by short cue-target intervals (Coull et al., 2000). Second, it has been found that temporal discrimination is better between 500 and 1000 ms than it is between 1000 and 1500 ms, with sounds beginning 500, 1000, and 1500 ms after the onset of a fixation point (as at the start of a trial; Sanders and Astheimer, 2008). Sanders and Astheimer (2008) found that this flexibility of temporally selective attention affects early perceptual processing.

**Table 2:** Comparison among PSS results.

	PSS			Difference		
Condition	Vol	Inv	No	Vol–Inv	Vol–No	Inv–No
Frissen et al., 2012	12	–45	4	^∗^	N.S.	^∗^
Nishi et al., 2014	5.3	–13.1	–37.7	^∗^	^∗∗^	^∗∗^
This study	14.5	–4.6	–9.8	^∗^	^∗^	N.S.

“Vol,” “Inv,” and “No” indicate voluntary, involuntary, and no movement conditions, respectively. “Vol–Inv,” “Vol–No,” and “Inv–No” indicate the differences between the respective conditions. A negative PSS represents the presentation of the tactile stimulus before the auditory stimulus. *p < 0.01; **p < 0.001.

**Table 2** also shows that voluntary movement shifts the PSS of an auditory–tactile TOJ from the tactile stimulus being first to the auditory stimulus being first (Vol–Inv and Vol–No columns), but that a proprioceptive sensation does not affect the PSS (Inv–No column). One possible explanation for the accelerated processing speed of the tactile stimulus by voluntary movement is efference copy. Efference copy, which is a copy of the motor command, is generated in the presupplementary motor cortex and the premotor cortex (Tanji and Mushiake, 1996). Evidence from three lines of research—functional magnetic resonance imaging (fMRI) experiments in humans (Cui et al., 2014), the activation of Brodmann area 2 (BA2) neurons in activity preceding the active movements of monkeys (Weber et al., 2011), and neurons recorded in the somatosensory cortex (SI, BA2 in particular) that only discharge during voluntary movements (London and Miller, 2013)—indicates that the efference copy can significantly influence the primary somatosensory cortices. The somatosensory cortex, which is also modulated by the premotor cortex during voluntary movements without proprioceptive feedback (Christensen et al., 2007), is an area of the brain that processes input from the various systems of the body, and is sensitive to touch. In addition, the efference copy is sent to the posterior parietal cortex (Desmurget et al., 2009), where tactile events are localized in external space (Azañon et al., 2010). Therefore, the efference copy of a voluntary movement may affect the processing speed of the tactile stimulus in the TOJ task used in this study.

A second possible explanation for the accelerated processing of the tactile stimulus in the voluntary movement condition is that the participants experienced the illusion of a self-generated tactile stimulus (as a kind of causal belief), which only occurs with self-paced voluntary movements. Based on this action-effect prediction (Waszak et al., 2012), the efference copies of the self-generated tactile stimulus and voluntary movement affected the processing speed of the tactile stimulus and then changed the PSS. This effect is identical to that in the Directions into Velocities of Articulators (DIVA) model in the online control of speech production. In the DIVA model, an efference copy of the motor command was found to be useful for motor preparation, and the auditory efference copy predicted the possible auditory outcome (Guenther et al., 2006). In addition, there is neurophysiological evidence of the human brain deploying efference copies in the somatosensory and auditory cortices in finger tapping and speech production tasks, respectively (Rauschecker and Scott, 2009; Tian and Poeppel, 2010).

In addition, voluntary movement may not only affect the processing speed of the tactile stimulus but also influence the TOJ task itself. Neural imaging evidence from fMRI studies has identified the activation of the temporal parietal junction (TPJ). This evidence was reported for TOJ tasks between two visual stimuli (Davis et al., 2009) and between two tactile stimuli (Takahashi et al., 2013), as well as between auditory and visual stimuli (Adhikari et al., 2013). The efference copy of a motor command is sent to the posterior parietal cortex (Desmurget et al., 2009), and the close relationship between the locations of the posterior parietal cortex and the TPJ proposed by Nishi et al. (2014) led us to infer that voluntary movement could influence the TOJ task itself.

Another reason why the shift of PSS occurring in voluntary movement (**Table 2**, Vol–Inv and Vol–No columns) may be related to the prior entry effect (Shore et al., 2001; Spence et al., 2001; Kitagawa et al., 2005; Zampini et al., 2005c). Both endogenous and exogenous attention to stimuli may change the PSS. In the present study, endogenous and exogenous attention may have been mixed. First, voluntary movement may enhance endogenous attention to tactile stimuli. The prior entry effect may have occurred and caused the PSS shift in the voluntary movement condition. Second, voluntary movement may decrease auditory exogenous attention, assuming that the auditory cue at the start of the trial increased auditory exogenous attention. We asked the participants to pay attention to a tactile stimulus in the three conditions to control for the prior entry effect (endogenous attention to tactile stimuli). However, voluntary movement may increase endogenous attention to tactile stimuli and decrease the effect of auditory exogenous attention. This attention shift may accelerate the speed of tactile processing and/or reduce the speed of auditory processing in the voluntary movement condition, which would lead to a PSS shift.

### Effect of Voluntary Movement on JND

**Table 3** shows the JND results of the previous and present studies. There were significant differences between the involuntary movement or no movement condition and the voluntary movement condition in Nishi et al.’s (2014) study, but there was no difference among the three conditions both in the present study and in Frissen et al.’s (2012) study. That is, both this study and Frissen et al.’s (2012) study failed to find an effect of voluntary movement on the JND.

**Table 3:** Comparison among JND results.

	JND			Difference		
Condition	Vol	Inv	No	Vol–Inv	Vol–No	Inv–No
Frissen et al., 2012	114	94	102	N.S.	N.S.	N.S.
Nishi et al., 2014	46.8	59.2	66.1	^∗^	^∗∗^	N.S.
This study	55.5	45.4	46.1	N.S.	N.S.	N.S.

“Vol,” “Inv,” and “No” indicate the voluntary, involuntary, and no movement conditions. “Vol–Inv,” “Vol–No,” and “Inv–No” indicate the differences between the respective conditions. *p < 0.01; **p < 0.001.

The present results suggest that the improved JND in Nishi et al.’s (2014) study were caused by the predictability of the stimulus. In their experiments, the tactile stimulus was always presented 500 ms after the finger movement in the voluntary movement condition. This could have allowed the participants to predict the occurrence of the stimulus and improve their JNDs (Petrini et al., 2009; Yokoyama et al., 2009; Vroomen and Stekelenburg, 2010). This stimulus predictability occurs only in the voluntary movement condition, because the JND in the involuntary movement condition, in which tactile stimulus was always presented 500 ms after the finger movement, did not differ from the JND in the no movement condition. On the other hand, the JND values in the present study are lower than those reported by Frissen et al. (2012). This means that the temporal window for auditory–tactile integration was narrower in this study than in the Frissen et al.’s (2012) study. We included a practice session in our experiment before the formal experimental trials to familiarize the participants with the TOJ task. Furthermore, the participants had additional practice in the voluntary movement condition to ensure appropriate finger speeds. Therefore, relative to the participants in Frissen et al.’s (2012) study, our participants were well-trained prior to the experimental conditions. The difference in JND values between the present study and Frissen et al.’s (2012) study was consistent with the findings of Hirsh and Sherrick (1961), in which well-trained participants performed better than less well-trained ones.

### Limitations

This study has some limitations. First, the practice session before the voluntary movement condition in which only tactile stimuli were presented may have an effect on the results (i.e., sensitization of the tactile channel in the voluntary movement condition). To eliminate the confounding effect of this practice session, the participants were given 2–3 min rest before each block of trials in the voluntary movement condition. We believe that this eliminated the effect of the voluntary movement condition practice runs on the observed results of the JND and PSS. First, according to a previous study (Hirsh and Sherrick, 1961), the more that people practice, the more their JNDs improve. However, JND did not improve in the voluntary movement condition in this study. This suggests that the potentially confounding effect of practice was well-controlled in this study. Second, according to another previous study (Zampini et al., 2005b), the amount of practice does not affect the PSS in auditory–tactile stimuli TOJ task. Therefore, there is no reason to believe that the practice session prior to the voluntary movement condition impacted the PSS result. However, further investigation may be necessary on this issue.

There may be a second limitation of this study related to stimulus intensity. Boenke et al. (2009) showed that stimulus intensity plays a role in the temporal perception of auditory–visual stimulus pairs. We used a stronger tactile stimulus in the present study than Frissen et al.’s (2012) study, and thus the strength of the tactile stimulus may have interacted strongly with the voluntary movement in our experiment. In future work, it would be interesting to investigate how the relationship between the strength of the tactile stimulus and voluntary movement affects simultaneous perception.

Finally, the ratio of male to female participants in this study was 5:1, which may limit the generalizability of the results. Although previous research has shown that there are no gender effect on two tactile TOJ task in the uncrossed arms condition (Cadieux et al., 2010) or on the temporal order threshold of two types of paired tones stimuli (Bao et al., 2013), it is unknown whether a gender difference exists in multimodal integration. Thus, it would be useful in future research to include more female participants to determine whether there is gender difference in the multimodal integration of auditory and tactile information in TOJ task.

## Conclusion

The purpose of this study was to investigate the effect of voluntary movement on auditory–tactile simultaneous perception, controlling for the effects of stimulus predictability, spatial information associated with large-scale movement, and other methodological problems (see Materials and Methods) found in previous studies (Kitagawa et al., 2009; Frissen et al., 2012; Nishi et al., 2014). Auditory–tactile TOJ tasks were conducted in voluntary, involuntary, and no movement conditions. The PSS in the voluntary movement condition shifted from the tactile stimulus being first in the involuntary movement or no movement condition to the auditory stimulus being first. JNDs did not differ across the three conditions. These results reveal that voluntary movement changes the PSS rather than the JND, but proprioceptive information does not affect the simultaneous perception of auditory and tactile stimuli.

Up until now, many studies of the simultaneous perception of multimodal information have focused on the no movement condition, in which participants simply receive information from the environment. However, we routinely act voluntarily on the environment and receive sensory feedback from the environment, with these two events together defining the moment. Therefore, it is necessary to study the simultaneous perception of multimodal information in voluntary movements, and not just in static (no movement) situations.

## Conflict of Interest Statement

The authors declare that the research was conducted in the absence of any commercial or financial relationships that could be construed as a potential conflict of interest.

## Abbreviations

JND: just noticeable difference
PSS: point of subjective simultaneity
SOAs: stimulus onset asynchronies
TOJ: temporal order judgment

## References

[B1] Adhikari B. M. Goshorn E. S. Lamichhane B. Dhamala M. (2013). Temporal-order judgment of audiovisual events involves network activity between parietal and prefrontal cortices. *Brain Connect.* 3 536–545. 10.1089/brain.2013.0163 23988147 PMC3796319

[B2] Azañon E. Longo M. R. Soto-Faraco S. Haggard P. (2010). The posterior parietal cortex remaps touch into external space. *Curr. Biol.* 20 1304–1309. 10.1016/j.cub.2010.05.063 20637619

[B3] Bao Y. Szymaszek A. Wang X. Oron A. Pöppel E. Szelag E. (2013). Temporal order perception of auditory stimuli is selectively modified by tonal and non-tonal language environments. *Cognition* 129 579–585. 10.1016/j.cognition.2013.08.019 24060605

[B4] Boenke L. T. Deliano M. Ohl F. W. (2009). Stimulus duration influences perceived simultaneity in audiovisual temporal order judgment. *Exp. Brain Res.* 198 233–244. 10.1007/s00221-009-1917-z 19590862

[B5] Cadieux M. L. Barnett-Cowan M. Shore D. I. (2010). Crossing the hands is more confusing for females than males. *Exp. Brain Res.* 204 431–446. 10.1007/s00221-010-2268-5 20574689

[B6] Cardoso-Leite P. Gorea A. Mamassian P. (2007). Temporal order judgment and simple reaction times: evidence for a common processing system. *J. Vis.* 7 1–14. 10.1167/7.6.11 17685794

[B7] Christensen M. S. Lundbye-Jensen J. Greertsen S. S. Petersen T. H. Paulson O. B. Nielsen J. B. (2007). Premotor cortex modulates somatosensory cortex during voluntary movements without proprioceptive feedback. *Nat. Neurosci.* 10 417–419. 17369825 10.1038/nn1873

[B8] Cohen J. (1988). *Statistical Power Analysis for the Behavioral Sciences* 2nd Edn Hillsdale, NJ: Erlbaum.

[B9] Coull J. Frith C. Büchel C. Nobre A. (2000). Orienting attention in time: behavioral and neuroanatomical distinction between exogenous and endogenous shifts. *Neuropsychologia* 38 808–819. 10.1016/S0028-3932(99)00132-3 10689056

[B10] Cui F. Arnstein D. Thomas R. M. Maurits N. M. Keysers C. Gazzola V. (2014). Functional magnetic resonance imaging connectivity analyses reveal efference-copy to primary somatosensory area, BA2. *PLoS ONE* 9:e84367 10.1371/journal.pone.0084367 PMC3885571 24416222

[B11] Davis B. Christie J. Rorden C. (2009). Temporal order judgments activate temporal parietal junction. *J. Neurosci.* 29 3182–3188. 10.1523/JNEUROSCI.5793-08.2009 19279255 PMC3862239

[B12] Desmurget M. Reilly K. T. Richard N. Szathmari A. Mottolese C. Sirigu A. (2009). Movement intention after parietal cortex stimulation in humans. *Science* 324 811–813. 10.1126/science.1169896 19423830

[B13] Finney D. J. (1952). *Probit Analysis: A Statistical Treatment of the Sigmoid Response Curve*. England: Cambridge University Press.

[B14] Frissen I. Ziat M. Campion G. Hayward V. Guastavino C. (2012). Effects of voluntary movements on auditory–haptic and haptic–haptic temporal order judgments. *Acta Psychol. (Amst.)* 141 140–148. 10.1016/j.actpsy.2012.07.010 22964054

[B15] Fujisaki W. Nishida S. (2009). Audio-tactile superiority over visuo-tactile and audio-visual combinations in the temporal resolution of synchrony perception. *Exp. Brain Res.* 198 245–259. 10.1007/s00221-009-1870-x 19499212

[B16] Guenther F. H. Ghosh S. S. Tourville J. A. (2006). Neural modeling and imaging of the cortical interactions underlying syllable production. *Brain Lang.* 96 280–301. 10.1016/j.bandl.2005.06.001 16040108 PMC1473986

[B17] Hermosillo R. Ritterband-Rosenbaum A. van Donkelaar P. (2011). Predicting future sensorimotor states influences current temporal decision making. *J. Neurosci.* 31 10019–10022. 10.1523/JNEUROSCI.0037-11.2011 21734293 PMC6703331

[B18] Hirsh I. J. Sherrick C. E. Jr. (1961). Perceived order in different sense modalities. *J. Exp. Psychol.* 62 423–432. 10.1037/h0045283 13907740

[B19] Kayser C. Petkov C. I. Logothetis N. K. (2008). Visual modulation of neurons in auditory cortex. *Cereb. Cortex* 18 1560–1574. 10.1093/cercor/bhm187 18180245

[B20] Keetels M. Vroomen J. (2005). The role of spatial disparity and hemifields in audio-visual temporal order judgments. *Exp. Brain Res.* 167 635–640. 10.1007/s00221-005-0067-1 16175363

[B21] Keetels M. Vroomen J. (2008). Temporal recalibration to tactile-visual asynchronous stimuli. *Neurosci. Lett.* 430 130–134. 10.1016/j.neulet.2007.10.044 18055112

[B22] Kitagawa N. Kato M. Kashino M. (2009). “Assessing the effect of voluntary action on sensitivity to temporal asynchrony between auditory and somatosensory events,” in *Poster Presented at the 10th International Multisensory Research Forum* New York.

[B23] Kitagawa N. Zampini M. Spence C. (2005). Audio-tactile interactions in near and far space. *Exp. Brain Res.* 166 528–537. 10.1007/s00221-005-2393-8 16091968

[B24] Kwon J. Ogawa K. Miyake Y. (2014). The effect of visual apparent motion on audiovisual simultaneity. *PLoS ONE* 9:e110224 10.1371/journal.pone.0110224 PMC4190322 25295594

[B25] Lewald J. Guski R. (2003). Cross-modal perceptual integration of spatially and temporally disparate auditory and visual stimuli. *Cogn. Brain Res.* 16 468–478. 10.1016/S0926-6410(03)00074-0 12706226

[B26] London B. M. Miller L. E. (2013). Responses of somatosensory area 2 neurons to actively and passively generated limb movements. *J. Neurophysiol.* 109 1505–1513. 10.1152/jn.00372.2012 23274308 PMC3774588

[B27] Miller J. Schwarz W. (2006). Dissociations between reaction times and temporal order judgments: a diffusion model approach. *J. Exp. Psychol. Hum. Percept. Perform.* 32 394–412. 10.1037/0096-1523.32.2.394 16634678

[B28] Mitrani L. Shekerdjiiski S. Yakimoff N. (1986). Mechanisms and asymmetries in visual perception of simultaneity and temporal order. *Biol. Cybern.* 54 159–165. 10.1007/BF00356854 3741893

[B29] Morrone M. C. Ross J. Burr D. (2005). Saccadic eye movements cause compression of time as well as space. *Nat. Neurosci.* 8 950–954. 10.1038/nn1488 15965472

[B30] Nijhawan R. (2002). Neural delays, visual motion and the flash-lag effect. *Trends Cogn. Sci.* 6 387–393. 10.1016/S1364-6613(02)01963-0 12200181

[B31] Nishi A. Yokoyama M. Ogawa K. Ogata T. Nozawa T. Miyake Y. (2014). Effects of voluntary movements on audio-tactile temporal order judgment. *IEICE Trans. Inf. Syst.* 6 1567–1573. 10.1109/SII.2011.6147525

[B32] Nishikawa N. Yasushi S. Makoto W. Nobutaka H. Kitazawa S. (2015). Effects of aging and idiopathic Parkinson’s disease on tactile temporal order judgment. *PLoS ONE* 10:e0118331 10.1371/journal.pone.0118331 PMC4356579 25760621

[B33] Petrini K. Russell M. Pollick F. (2009). When knowing can replace seeing in audiovisual integration of actions. *Cognition* 110 432–439. 10.1016/j.cognition.2008.11.015 19121519

[B34] Rauschecker J. Scott S. (2009). Maps and streams in the auditory cortex: nonhuman primates illuminate human speech processing. *Nat. Neurosci.* 12 718–724. 10.1038/nn.2331 19471271 PMC2846110

[B35] Sanders L. D. Astheimer L. B. (2008). Temporally selective attention modulates early perceptual processing: event-related potential evidence. *Percept. Psychophys.* 70 732–742. 10.3758/PP.70.4.732 18556935 PMC2676724

[B36] Schneider K. A. Bavelier D. (2003). Components of visual prior entry. *Cogn. Psychol.* 47 333–366. 10.1016/S0010-0285(03)00035-5 14642288

[B37] Shi Z. Hirche S. Schneider W. Müller H. (2008). “Influence of visuomotor action on visual-haptic simultaneous perception: a psychophysical study,” in “ *Proceeding of the Symposium on Haptic Interfaces for Virtual Environments and Teleoperator Systems* (Reno, NE: IEEE) 65–70.

[B38] Shore D. I. Spence C. Klein R. M. (2001). Visual prior entry. *Psychol. Sci.* 12 205–212. 10.1111/1467-9280.00337 11437302

[B39] Slutsky D. A. Recanzone G. H. (2001). Temporal and spatial dependency of the ventriloquism effect. *Neurol. Rep.* 12 7–10. 10.1097/00001756-200101220-00009 11201094

[B40] Spence C. Shore D. I. Klein R. M. (2001). Multisensory prior entry. *J. Exp. Psychol. Gen.* 130 799–832. 10.1037/0096-3445.130.4.799 11757881

[B41] Takahashi T. Kansaku K. Wada M. Shibuya S. Kitazawa S. (2013). Neural correlates of tactile temporal-order judgment in humans: an fMRI study. *Cereb. Cortex* 23 1952–1964. 10.1093/cercor/bhs179 22761307

[B42] Tanji J. Mushiake H. (1996). Comparison of neuronal activity in the supplementary motor area and primary motor cortex. *Cogn. Brain Res.* 3 143–150. 10.1016/0926-6410(95)00039-9 8713555

[B43] Tian X. Poeppel D. (2010). Mental imagery of speech and movement implicates the dynamics of internal forward models. *Front. Psychol.* 1:166 10.3389/fpsyg.2010.00166 PMC3158430 21897822

[B44] Van Eijk R. L. J. Kohlrausch A. Juola J. F. van de Par S. (2009). Temporal interval discrimination thresholds depend on perceived synchrony for audio-visual stimulus pairs. *J. Exp. Psychol. Hum. Percept. Perform.* 35 1254–1263. 10.1037/a0014254 19653763

[B45] Vogels I. M. (2004). Detection of temporal delays in visual-haptic interfaces. *Hum. Factors* 46 118–134. 10.1518/hfes.46.1.118.30394 15151159

[B46] Vroomen J. Stekelenburg J. J. (2010). Visual anticipatory information modulates multisensory interactions of artificial audiovisual stimuli. *J. Cogn. Neurosci.* 22 1583–1596. 10.1162/jocn.2009.21308 19583474

[B47] Waszak F. Cardoso-Leite P. Hughes G. (2012). Action effect anticipation: neurophysiological basis and functional consequences. *Neurosci. Biobehav. Rev.* 36 943–959. 10.1016/j.neubiorev.2011.11.004 22108008

[B48] Weber D. J. London B. M. Hokanson J. A. Ayers C. A. Gaunt R. A. Torres R. R. (2011). Limb-state information encoded by peripheral and central somatosensory neurons: implications for an afferent interface. *IEEE Trans. Neural Syst. Rehabil. Eng.* 19 501–513. 10.1109/TNSRE.2011.2163145 21878419 PMC3694199

[B49] Wenke D. Haggard P. (2009). How voluntary actions modulate time perception. *Exp. Brain Res.* 196 311–318. 10.1007/s00221-009-1848-8 19471909 PMC2700248

[B50] Yarrow K. Haggard P. Heal R. Brown P. Rothwell J. C. (2001). Illusory perceptions of space and time preserve cross-saccadic perceptual continuity. *Nature* 414 302–305. 10.1038/35104551 11713528

[B51] Yokoyama M. Yoshida S. Ora H. Miyake Y. (2009). “Effect of voluntary motion in inter-modal simultaneity judgment,” in *Proceedings of Human Interface Symposium* Tokyo 877–882.

[B52] Zampini M. Brown T. Shore D. I. Maravita A. Röder B. Spence C. (2005a). Audiotactile temporal order judgments. *Acta Psychol.* 118 277–291. 10.1016/j.actpsy.2004.10.017 15698825

[B53] Zampini M. Guest S. Shore D. I. Spence C. (2005b). Audio-visual simultaneity judgments. *Percept. Psychophys.* 67 531–544. 10.3758/BF03193329 16119399

[B54] Zampini M. Shore D. I. Spence C. (2005c). Audiovisual prior entry. *Neurosci. Lett.* 381 217–222. 10.1016/j.neulet.2005.01.085 15896473

[B55] Zampini M. Shore D. I. Spence C. (2003). Audiovisual temporal order judgments. *Exp. Brain Res.* 152 198–210. 10.1007/s00221-003-1536-z 12879178

